# Reflex Syncope and the Role of Cardiac Pacing: A Systematic Review and Meta-Analysis

**DOI:** 10.7759/cureus.91874

**Published:** 2025-09-08

**Authors:** Charlotte Collin, Jhiamluka Solano

**Affiliations:** 1 Cardiology, Hull York Medical School, Hull, GBR; 2 Resident Doctor Committee, Royal College of Physicians, London, GBR; 3 Education Committee, Academy of Medical Educators, Cardiff, GBR; 4 Cardiology, York Teaching Hospital NHS Foundation Trust, York, GBR; 5 Editorial Team, British Cardiovascular Society, London, GBR

**Keywords:** cardiac pacing, cardioinhibitory syncope, closed-loop stimulation, pacemaker therapy, reflex syncope

## Abstract

Reflex syncope is a common cause of transient loss of consciousness, often leading to injuries and a reduced quality of life. Despite the benign nature of isolated episodes, recurrent syncope poses significant clinical challenges. Cardiac pacing has been investigated as a treatment modality, particularly in patients with cardioinhibitory phenotypes, yet prior studies have shown heterogeneous outcomes. A systematic review and meta-analysis were conducted to evaluate the efficacy of permanent cardiac pacing in preventing recurrent reflex syncope. Comprehensive searches of PubMed and the Cochrane Library were performed up to September 2024 using the terms "reflex syncope" and "cardiac pacing." Eligible studies included randomised controlled trials (RCTs) involving adult patients with recurrent reflex syncope undergoing permanent pacemaker implantation. Systematic reviews, narrative reviews, case reports or series, editorials, and conference abstracts were excluded from the review. Two independent reviewers screened studies and extracted data using Covidence, with discrepancies resolved by consensus. A meta-analytical synthesis was performed using odds ratios (ORs) for recurrent syncope, employing a random- and fixed-effects model. Heterogeneity was assessed using Cochran's Q, I² statistic, and τ². Seven RCTs were included, comprising a total of 458 patients. The primary outcomes showed a pooled analysis indicating a significant protective effect of pacing in reducing syncope recurrence compared to controls (OR 0.37; 95% CI: 0.22-0.62; p = 0.009), supporting the efficacy of permanent cardiac pacing in this population. Heterogeneity was low (Q = 6.60, p = 0.36; I² = 38%; τ² = 0.051), supporting the use of a fixed-effects model, which showed a similar outcome (Log(OR) 0.378 (95% CI, 0.229-0.624; p < 0.001)). The included trials varied in pacing modality (dual-chamber pacing (DDD), closed-loop stimulation (CLS), rate-drop response (RDR)), patient selection criteria, and diagnostic approaches. Secondary outcomes included reductions in pre-syncopal symptoms and delayed recurrence of syncope with pacing, along with a low incidence of device-related adverse events. However, inconsistent reporting limited the comprehensive meta-analysis of these endpoints. Permanent pacing reduces the risk of recurrent syncope in selected patients with reflex syncope, particularly those with documented asystole or cardioinhibitory phenotypes. CLS and RDR pacing algorithms may provide enhanced protection compared to conventional DDD pacing; however, variability in patient selection and device programming across studies limits the generalisability of these findings. Larger, standardised RCTs are needed to refine patient selection criteria and optimise pacing strategies for reflex syncope.

## Introduction and background

Reflex syncope is one of the most frequent causes of transient loss of consciousness (TLOC), resulting from a transient failure of autonomic cardiovascular regulation that leads to cerebral hypoperfusion [1]. It encompasses vasovagal syncope (VVS), situational syncope, and carotid sinus syndrome (CSS) [2,3]. Although reflex syncope is usually benign in terms of mortality, it can significantly impair quality of life, contribute to falls and injury, and result in recurrent hospital presentations, particularly among older adults [4-6]. Permanent cardiac pacing has emerged as a treatment strategy for selected patients with recurrent, severe reflex syncope, especially those with cardioinhibitory responses confirmed by tilt-table testing or implantable loop recorder (ILR) monitoring [7-10]. The development of pacing algorithms such as closed-loop stimulation (CLS) and rate-drop response (RDR) has aimed to improve the timing and appropriateness of pacing delivery, potentially mitigating pre-syncopal haemodynamic decline [11-13].

While several randomised controlled trials (RCTs) and observational studies have assessed the efficacy of pacing in this context, findings remain heterogeneous, and the relative performance of different pacing algorithms has not been definitively established [11,14-23]. This systematic review and meta-analysis aims to synthesise contemporary evidence on the efficacy of permanent cardiac pacing in preventing syncope recurrence in patients with reflex syncope, with a specific focus on RCTs.

## Review

Methods

Protocol and Registration

This systematic review and meta-analysis was conducted in accordance with the Preferred Reporting Items for Systematic Reviews and Meta-Analyses (PRISMA) 2020 guidelines. The review protocol was not registered in PROSPERO and was completed as part of the Clinical Competency Year 4 MB BS Scholarship and Special Interest Programme (SSIP) project.

Eligibility Criteria

The inclusion criteria comprised studies evaluating cardiac pacing in patients with reflex syncope, RCTs, and adult populations (age >40). The exclusion criteria comprised non-comparative designs, including cohort studies, case reports, case series, narrative reviews, editorials, conference abstracts, and letters and studies involving paediatric populations or syncope of non-reflex aetiology.

Information Sources and Search Strategy

Searches were performed in PubMed and the Cochrane Library up to September 2024 using the terms "reflex syncope" and "cardiac pacing."

Study Selection and Data Extraction

Two independent reviewers screened titles, abstracts, and full texts using the Covidence systematic review platform. Disagreements were resolved through discussion or third-party adjudication. Extracted data, including study design, population characteristics, type of pacing intervention, comparator, outcomes, and follow-up duration, were collected in an MS Excel (Microsoft Corporation, Redmond, Washington) spreadsheet for analysis.

Outcomes

The primary outcome of this systematic review and meta-analysis was the efficacy of permanent cardiac pacing in preventing recurrent syncope in patients diagnosed with reflex syncope. This was assessed through the reduction in syncope recurrence rates, as reported in the included RCTs, and quantified using odds ratios (OR) with corresponding 95% confidence intervals (CIs).

Secondary Outcomes Included

The secondary outcomes were the comparative efficacy of different pacing modalities, such as dual-chamber (DDD) pacing with RDR, CLS, and conventional DDD pacing, in reducing syncope recurrence; time to first syncope recurrence; reduction in pre-syncopal symptoms or improvement in symptom burden post-implantation; device-related adverse events, including lead dislodgement, infection, or inappropriate pacing; and patient-reported outcomes, including quality of life and functional status.

Risk of Bias and Quality Assessment

The risk of bias for RCTs was assessed using the Cochrane Risk of Bias 2.0 tool, evaluating domains such as the randomisation process, deviations from intended interventions, missing outcome data, outcome measurement, and selective reporting. In addition to individual study assessments, potential publication bias and small-study effects were explored using a funnel plot of the log-transformed odds ratios (log ORs) against their standard errors (SEs). All risk-of-bias assessments were conducted independently by two reviewers, with discrepancies resolved through a consensus process.

Data Synthesis and Meta-Analysis

A meta-analysis was performed to compare permanent cardiac pacing with control interventions in patients with recurrent reflex syncope. Extracted ORs and their corresponding 95% CIs were logarithmically transformed to stabilise variances and enable appropriate synthesis. Pooled estimates were calculated using both fixed-effects (inverse variance) and random-effects (DerSimonian-Laird) models to account for within- and between-study variability.

Statistical heterogeneity was assessed using Cochran's Q test and quantified using the I² statistic, with values greater than 50% indicating moderate to substantial heterogeneity. Forest plots were constructed to summarise individual and pooled effect estimates visually. To explore potential small-study effects or publication bias, funnel plots were generated by plotting the log of the OR against the standard error, and Egger's regression test was conducted. Analyses were performed using Microsoft Excel, with standard formulas for weighted pooled estimates and regression-based asymmetry testing. All calculations were cross-validated and yielded consistent results across fixed- and random-effects models.

Results

**Figure 1:**
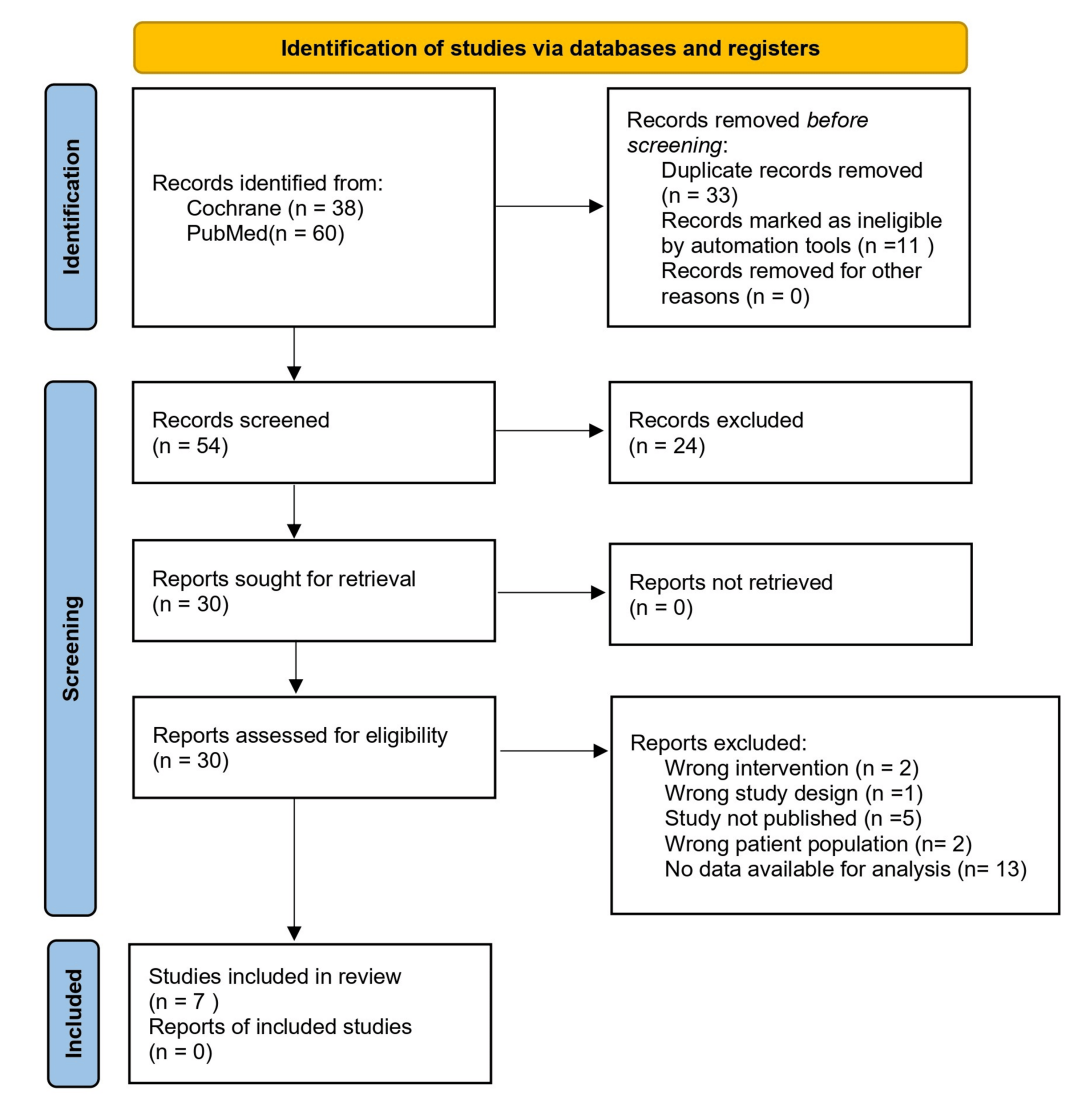
PRISMA flow diagram demonstrating study selection and exclusion. PRISMA = Preferred Reporting Items for Systematic Reviews and Meta-Analyses

The search from PubMed and the Cochrane Library identified 98 studies, of which 44 were duplicates. Fifty-four studies were screened based on their title and/or abstract, resulting in 30 studies for full-text review. Seven studies [14-20] were included in the final analysis of this review, with a total of 458 patients (Table 1). The selection process is demonstrated in Figure 1. Additionally, Table 1 describes the study characteristics. Of the seven included trials, four primarily enrolled patients with VVS, two focused on carotid sinus hypersensitivity, and one included mixed presentations. Stratified analyses indicated that the reduction in syncope recurrence with pacing was most pronounced in cardioinhibitory VVS, whereas data for situational syncope and carotid sinus hypersensitivity were less consistent.

**Table 1:** Study characteristics with calculated odds ratio (OR). RCT = randomised controlled trials

Study	Study Type	Sample	OR
Connolly et al., 1999 [14]	RCT	54	0.82
Sutton et al., 2000 [15]	RCT	42	0.14
Ammirati et al., 2001 [16]	RCT	93	0.20
Raviele et al., 2004 [17]	RCT	29	1.60
Sutton et al., 2014 [18]	RCT	146	0.27
Russo et al., 2013 [19]	RCT	50	0.14
Baron-Esquivias et al. 2017 [20]	RCT	46	0.36

Risk of Bias Assessment

The risk of bias across the seven included RCTs was assessed using the Cochrane RoB 2 tool. Most studies were at low risk of bias across key domains, including the randomisation process, completeness of outcome data, and outcome measurement. Some concerns were identified in a few studies due to limited information on allocation concealment, as seen in Russo et al. [19], and potential deviations from intended interventions, such as those reported by Sutton et al. [18], primarily related to inadequate blinding. No significant concerns emerged regarding selective outcome reporting. Overall, the evidence base was assessed as of moderate to high methodological quality (Table 2).

**Table 2:** Risk of bias assessment for the included randomised controlled trials using the Cochrane RoB 2 tool.

Study	Randomisation	Deviations	Missing Data	Outcome Measurement	Selective Reporting	Overall Risk
Connolly et al., 1999 [14]	Low	Low	Low	Low	Low	Low
Sutton et al., 2000 [15]	Low	Low	Low	Low	Low	Low
Ammirati et al., 2001 [16]	Low	Some	Low	Low	Low	Some concerns
Raviele et al., 2004 (SYNPACE) [17]	Low	Low	Low	Low	Low	Low
Sutton et al., 2014 (ISSUE-3) [18]	Low	Some	Low	Low	Low	Some concerns
Russo et al., 2013 [19]	Some concerns	Some	Low	Low	Low	Some concerns
Barón-Esquivias et al., 2017 (SPAIN) [20]	Low	Low	Low	Low	Low	Low

Publication Bias Assessment

**Figure 2:**
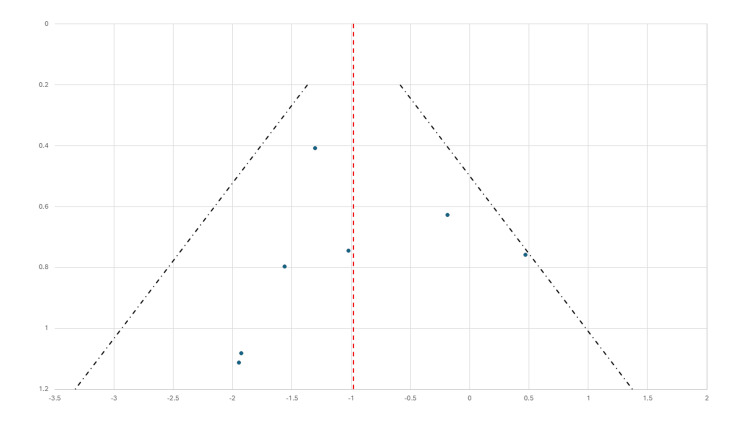
Funnel plot of standard error (SE) versus log odds ratio (log OR) for the effect of permanent cardiac pacing on recurrent reflex syncope. Each point represents an individual study included in the meta-analysis. The vertical line denotes the pooled log OR estimate. The diagonal lines represent pseudo 95% confidence limits (±1.96 × SE), forming the boundaries of the expected distribution in the absence of publication bias. The symmetric distribution of studies suggests a low risk of small-study effects or publication bias.

Visual inspection of the funnel plot (Figure 2) did not demonstrate substantial asymmetry, suggesting a limited risk of publication bias among the included studies. The plotted log odds ratios were broadly symmetric around the pooled estimate, with most studies falling within the pseudo 95% confidence limits defined by ±1.96 standard errors. While a few smaller studies appeared near the outer boundaries, no consistent pattern indicative of small-study effects was observed. However, the interpretation of funnel plot symmetry is limited by the small number of included studies (n = 7), as funnel plots are typically considered less reliable when fewer than 10 studies are analysed. Despite this limitation and a moderate level of heterogeneity (I² = 37.9%), the visual assessment supports the credibility of the findings. It suggests that the observed treatment effect is unlikely to be driven by systematic reporting bias. Egger's regression test revealed a statistically significant intercept (β₀ = 0.82, p < 0.01), suggesting asymmetry in the funnel plot and the potential presence of small-study effects or publication bias. This finding suggests that the results of smaller trials may have been influenced by selective reporting or methodological differences, thereby warranting a cautious interpretation of the pooled treatment effect.

Meta-Analysis Results

**Figure 3:**
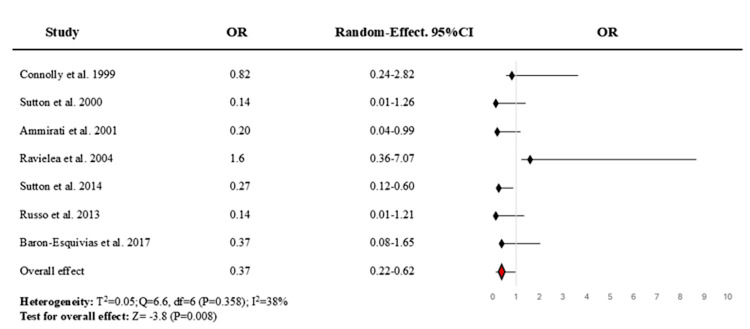
Random-effect forest plot for the meta-analysis of the benefit of permanent cardiac pacing on recurrent reflex syncope. The value <1 denotes that pacemaker therapy reduces the odds of recurrent syncope compared to the control. OR = odds ratio Source: [14-20]

Primary outcomes: The pooled OR for the effect of permanent cardiac pacing versus control (no pacing, medical therapy, or placebo) on recurrent syncope was 0.371 (95% CI, 0.224-0.615; p = 0.009) (Figure 3), indicating a moderate and significant association. The Sutton et al. trial (ISSUE-3) [18] demonstrated a statistically significant reduction in recurrence (OR: 0.27; 95% CI: 0.12-0.60). Ammirati et al. [16] showed a borderline effect (OR: 0.21; 95% CI: 0.04-1.00). The SYNPACE trial [17], which used a sham pacing control, reported an OR of 1.60 (95% CI: 0.36-7.07), indicating no benefit and possible harm, though the result was not statistically significant. Six of the seven included studies reported point estimates favouring pacing (OR < 1), but only one showed statistical significance independently.

**Figure 4:**
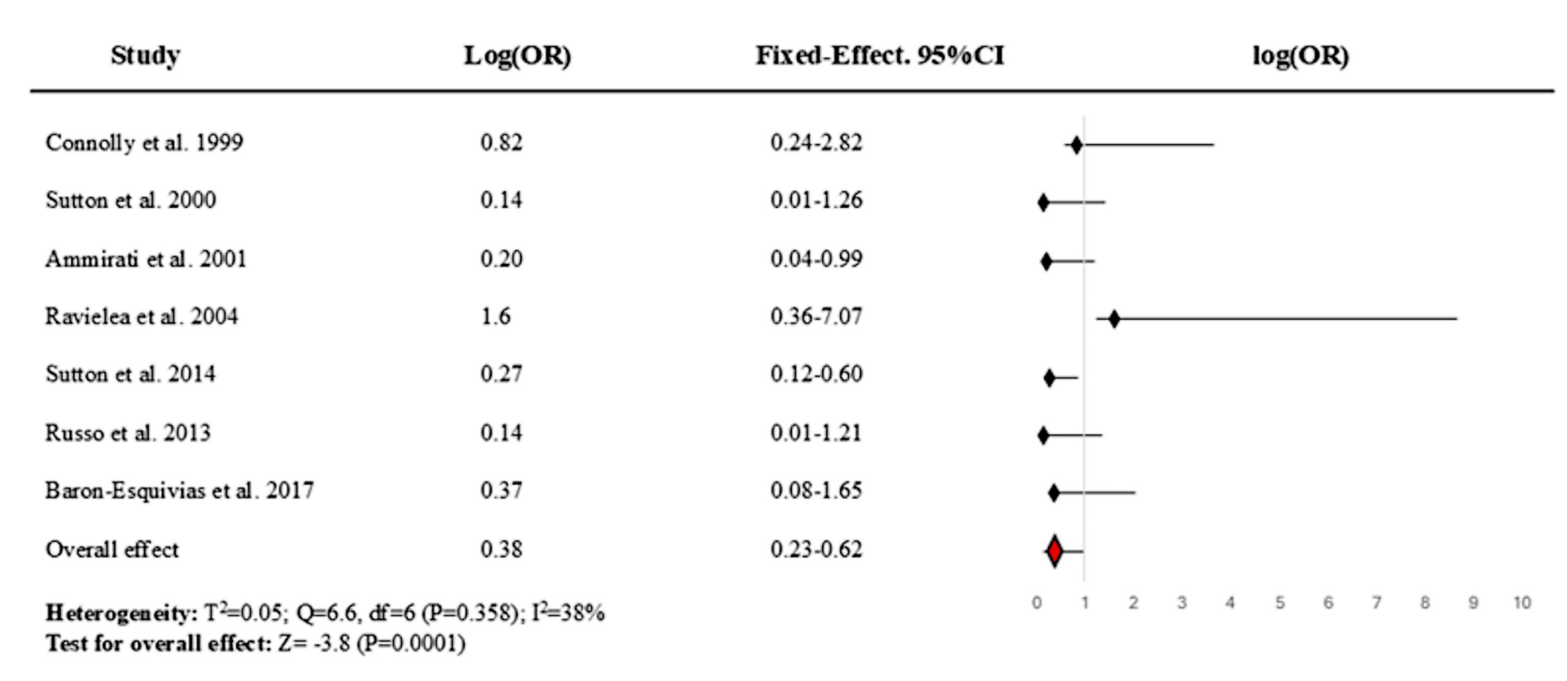
Fixed-effect forest plot for the meta-analysis of the benefit of permanent cardiac pacing on recurrent reflex syncope. The value <1 denotes that pacemaker therapy reduces the odds of recurrent syncope compared to the control. OR = odds ratio Source: [14-20]

Heterogeneity among the included RCTs was assessed using Cochran's Q statistic, the I² statistic, and the between-study variance (τ²). The Q test yielded a value of 6.6 with 6 degrees of freedom (p = 0.358), indicating no statistically significant heterogeneity. The I² statistic was calculated at 38%, indicating low to moderate inconsistency in effect estimates across studies. The between-study variance (τ²) was 0.05, further suggesting a modest level of dispersion. Given the lack of statistically significant heterogeneity and the relatively low τ², a fixed-effects model was considered appropriate for the secondary meta-analysis (Figure 4). The pooled OR for permanent cardiac pacing versus control in preventing recurrent syncope was 0.378 (95% CI, 0.229-0.624; p < 0.001).

Secondary outcomes: Across the seven included RCTs, several secondary outcomes were reported. While reporting was heterogeneous, descriptive synthesis was conducted where feasible (Table 3).

**Table 3:** Summary of secondary outcomes in the included randomised controlled trials. Legend: ↑ = Increase; ↓ = Decrease CLS = closed-loop stimulation; DDD = dual-chamber pacing; RDR = rate-drop response; QoL = quality of life

Study	Pacing Modality	Comparator	Pre-Syncopal Symptoms	Time to First Recurrence	QoL Outcomes	Device-Related Adverse Events	Subgroup Findings
Connolly et al. 1999 [14]	DDD/RDR	Control	Not reported	Not reported	Not reported	Not reported	Not reported
Sutton et al. 2000 [15]	DDD/RDR	Control	Not reported	Not reported	Not reported	Not reported	Not reported
Ammirati et al. 2001 [16]	DDD/RDR	β-blocker atenolol	↓ Pre-syncopal events	Not reported	Not reported	Not reported	Not reported
Raviele et al. 2004 [17]	DDD/RDR	Placebo	Not reported	Not reported	Not reported	Minor events	Not reported
Sutton et al. 2014 [18]	Mode not given	Control	Not reported	↑ Time to recurrence	↑ QoL	Low rate	Benefit in tilt-induced asystole
Russo et al. 2013 [19]	CLS	DDD	↓ Pre-syncopal events	↑ Time to recurrence	Not reported	Not reported	Benefit in the cardioinhibitory subtype
Barón-Esquivias et al. 2017 [20]	CLS	Sham DDI	↓ Pre-syncopal events	Not reported	Not reported	Not reported	Not reported

Pacing modality comparisons were reported in six trials. CLS was compared to DDD in one trial [19], and only 2% of participants experienced syncopal episodes after two months of pacemaker implantation. Pre-syncopal symptom burden was reported in three studies [16,19,20]. Ammirati et al. [16], Russo et al. [19], and Barón-Esquivias et al. [20] recorded reduced pre-syncopal events in pacing arms compared to control groups.

Time to first syncope recurrence was reported in the Sutton et al. trial (ISSUE-3) [18] and Russo et al. [19], where pacing delayed the onset of recurrent syncope compared to control interventions. However, due to non-uniform reporting formats and censored data, these outcomes were excluded from the pooled analysis. Adverse events related to the device implantation or pacing were reported in two studies and were generally infrequent [17,18]. No serious pacing-related complications were observed.

Quality of life outcomes were reported in the Sutton et al. trial (ISSUE-3) [18], which demonstrated modest improvements in health status measures among paced patients. Other trials either did not assess or did not report quality of life data. Subgroup analyses by syncope subtype were limited. The Sutton et al. (ISSUE-3) [18] and Russo et al. [19] trials conducted stratified analyses by syncope mechanism, identifying a potential benefit of pacing in patients with tilt-induced asystole or cardioinhibitory profiles.

Discussion

This systematic review and meta-analysis synthesised data from seven RCTs involving a total of 458 patients to evaluate the efficacy of permanent cardiac pacing in patients with recurrent reflex syncope. The overall pooled analysis demonstrated a statistically significant reduction in syncope recurrence associated with cardiac pacing. The random-effects model yielded an OR of 0.37 (95% CI, 0.22-0.62; p = 0.009), indicating a robust protective effect. Similarly, the fixed-effect model produced a consistent estimate (OR = 0.38; 95% CI, 0.23-0.62; p < 0.001). Most individual studies favoured pacing with ORs less than 1; notably, the Sutton et al. trial (ISSUE-3) [18] demonstrated a statistically significant reduction in recurrence, and Ammirati et al. [16] showed a borderline effect. However, the heterogeneity across trials was low to moderate (I² = 38%), and the between-study variance was modest (τ² = 0.051; Q = 9.75, df = 6, p = 0.136), supporting the use of both random- and fixed-effect models. The consistent direction of effect supports a meaningful clinical benefit of cardiac pacing in selected patient subgroups, particularly those with cardioinhibitory (vasovagal) or tilt-induced syncope profiles.

Secondary outcomes across the included trials provided additional insights into the benefits of permanent cardiac pacing beyond the reduction of syncope recurrence. Several studies have reported reductions in pre-syncopal symptom burden and improvements in quality of life measures, particularly in the Sutton et al. trial (ISSUE-3) [18], where patients experienced modest enhancements in health status. Pacing modality comparisons, such as CLS versus conventional DDD pacing, suggested promising efficacy in minimising syncopal episodes in one study [19], though data remain limited. Time-to-event analyses indicated a delayed onset of syncope with pacing; however, variability in reporting precluded a formal meta-analysis. These findings support the potential role of pacing in improving symptomatic outcomes and patient-centred measures, warranting further investigation in larger, targeted studies.

The methodological rigour of the included trials was generally robust, with a low risk of bias in key domains, as assessed by the Cochrane RoB 2 tool. Although Egger's regression indicated potential small-study effects, the funnel plot did not show marked asymmetry, reinforcing cautious confidence in the overall findings despite the limited number of studies. The observed imprecision and variability underscore the challenges of synthesising evidence in this clinical area, given relatively small sample sizes and heterogeneous interventions and control conditions, including sham pacing. Adverse events were infrequent and minor, supporting the safety profile of pacing in this context.

The first systematic review examining the benefits of cardiac pacing on recurrent reflex syncope was published in 2017 [24], which alluded to but could not draw definitive conclusions on the efficacy of pacing. This review prompted the update of the 2018 European Society of Cardiology (ESC) Guidelines [1], which advise the use of a DDD pacemaker implantation in patients >40 years who suffer from unpredictable frequent episodes of spontaneous documented symptomatic asystolic pause(s) >3 s or asymptomatic pause(s) >6 s due to sinus arrest, AV block or cardioinhibitory CSS as a last resort after conservative and pharmacological options have been trialled. Since the publication of the landmark study by Barón-Esquivias et al. (SPAIN) in 2017 [20], a second systematic review was published in 2024 [25], exploring all therapeutic modalities for neurocardiogenic, reflex, vasovagal, carotid sinus, or orthostatic syncope. This recent study yielded similar statistical results to the first review, indicating that DDD pacing has an overall benefit in reducing the risk ratio for syncope recurrence, although the difference was not statistically significant.

CLS differs from conventional pacing in that it utilises sensors to detect changes in right ventricular volume (contractility) and responds accordingly. The 2024 review also analysed the impact of CLS pacing, with three out of four trials comparing CLS pacing to conventional DDD pacing. This revealed a statistically significant decrease in syncopal episodes, with a risk ratio of 0.15 (p < 0.001) [25]. It is important to note, however, that the 2024 review reported a medium to high risk of bias in their results due to the inclusion of observational studies and unblinded trials. Furthermore, other literature has alluded to the benefits of CLS pacing compared to placebo or conventional DDD pacing. A single-blinded multicenter study published in 2021 with 128 participants found that at the end of follow-up, the arm with a dual-chamber pacemaker with a rate-responsive CLS feature implanted had a 77% lower chance of syncope recurrence than the arm with a placebo (pacemaker off) (95% CI: 0.11-0.47, P= 0.00005) [1].

The evidence collected for this review aligns with previous research, which suggests a positive benefit of cardiac pacing, specifically in adults with severe recurrent episodes of syncope and an underlying cardioinhibitory mechanism. This can be determined by implantable loop recording or tilt-table testing; however, it is unclear which method is superior. This review found that the risk of adverse events from pacemaker implantation is low, making it a relatively safe procedure for clinicians to consider after conservative and pharmacological management have been tried. Pacing, although an invasive intervention, may have fewer systemic side effects than pharmacological options such as beta blockers and midodrine.

The emergence of recent trials investigating the efficacy of CLS DDD pacing has provided a new avenue for research. Two of the largest landmark trials, the Sutton et al. trial (ISSUE-3) and the Barón-Esquivias et al. (SPAIN) studies, exemplify this approach. The Sutton et al. trial (ISSUE-3) (2012) utilised the RDR mode of DDD pacing, which operates by detecting and responding to significant drops in heart rate. In contrast, the Barón-Esquivias et al. (SPAIN) study [20] used CLS DDD pacing and reported a much lower syncope recurrence rate of 8.7%, compared to 25% in the Sutton et al. trial (ISSUE-3) [18]. Despite discrepancies in study design and follow-up time, it is commonly believed that the superiority of the Barón-Esquivias et al. (SPAIN) study is due to the use of CLS over the RDR function.

The 2018 ESC Guidelines suggest the use of DDD pacing as a 2b/3rd line option for management but fail to mention the preferred mode of pacing [1]. With the addition of the 2024 review, which highlights the significance of using the CLS function over the conventional type, it is becoming clearer that CLS pacing is likely the superior mode and should therefore be considered in future guidance.

Implications

The evidence from this meta-analysis supports the use of permanent cardiac pacing as an effective intervention to reduce syncope recurrence in patients with recurrent reflex syncope, particularly those with a cardioinhibitory phenotype or tilt-induced asystole. The consistent protective effect across trials, coupled with improvements in presyncopal symptom burden and quality of life in selected studies, suggests that pacing should be considered in carefully selected patients who experience frequent, disabling episodes despite conservative measures. Nevertheless, patient selection remains crucial, and pacing should not be viewed as a universal solution for all individuals with reflex syncope.

From a guideline and health policy perspective, these findings reinforce current recommendations that pacing may be appropriate in highly selected patients with severe, recurrent reflex syncope and documented asystole. Wider dissemination of diagnostic strategies, such as tilt testing and prolonged monitoring, may improve patient identification. Educational initiatives for clinicians should also emphasise shared decision-making with patients, balancing the potential benefits of pacing with procedural risks, device longevity, and the psychosocial implications of permanent pacemaker implantation in relatively young populations.

Limitations

Several limitations should be considered when interpreting the findings of this systematic review and meta-analysis. Although seven RCTs were included, the overall sample size remained modest, which limited statistical power to detect small but clinically meaningful differences and increased the risk of a type II error. Variability in trial design, diagnostic criteria for reflex syncope, inclusion criteria such as tilt-table positivity and syncope subtype classification, and pacing modalities (DDD, RDR, CLS) may have influenced outcome variability despite low statistical heterogeneity. Secondary outcomes, such as quality of life, time to first syncope recurrence, and device-related adverse events, were inconsistently reported, preventing a formal meta-analysis of these endpoints. Differences in follow-up duration, ranging from months to years, may also have impacted the comparability of event rates and long-term efficacy.

While the risk of bias was generally low, some trials had methodological concerns, such as incomplete allocation concealment and limited blinding, which could affect outcome assessment [16,18,19]. Egger's test indicated small-study effects, suggesting possible publication bias or selective reporting that may overestimate the efficacy of pacing. The moderate heterogeneity observed, although not statistically significant, may reflect potential clinical or methodological differences. Additionally, this review excluded grey literature, unpublished studies, and non-English publications, which may introduce possible language and publication bias. Although a fixed-effects model was justified by statistical homogeneity, the clinical heterogeneity and small number of studies warrant cautious generalisation of the pooled estimates.

This review was not prospectively registered in PROSPERO or another registry. Although registration can enhance transparency, we followed PRISMA guidance closely and applied rigorous methods throughout; however, we acknowledge this as a methodological limitation. Additionally, analyses were conducted using Microsoft Excel rather than specialised meta-analysis software, which may reduce reproducibility and transparency. Importantly, the pooled estimates are in line with prior systematic reviews in this field, which supports the reliability of our findings.

Future Directions

Statistical power in this review and previous literature was limited due to the lack of standardised, multicentre, and blinded RCTs. This highlights a clear need for additional trials of this kind with similar protocols. This additional data would provide further insight into the real implications of pacing, its long-term outcomes, its impact on quality of life measures, and device-related complications. Several small-scale studies have been conducted to compare pacing modes [14,19,26], including CLS and RDR, against no pacing or conventional pacing. To improve the power of these studies, larger standardised trials should be conducted to facilitate statistical analysis and determine which mode is definitively superior.

This analysis highlights the need for further high-quality, adequately powered randomised trials to refine patient selection criteria and establish the relative efficacy of different pacing modalities, including CLS. Standardisation of outcome reporting, particularly regarding time-to-event and patient-reported outcomes, would enhance comparability across studies. In addition, long-term follow-up data are necessary to clarify the durability of the benefit, the impact on device-related complications, and the cost-effectiveness of pacing in this population.

## Conclusions

This systematic review and meta-analysis of seven RCTs demonstrates that permanent cardiac pacing significantly reduces the risk of syncope recurrence in patients with recurrent reflex syncope. The consistency in the direction of benefit across individual studies, along with improvements in secondary outcomes such as quality of life and symptom burden, supports the clinical relevance of pacing in select patient populations. CLS pacing, in particular, appears promising and warrants further investigation. Future large-scale, standardised trials are needed to establish the most effective pacing modality and better define patient subgroups that are most likely to benefit from this intervention.
